# Effect of postextubation high-flow nasal cannula therapy on lung recruitment and overdistension in high-risk patient

**DOI:** 10.1186/s13054-020-2809-7

**Published:** 2020-03-06

**Authors:** Rui Zhang, Huaiwu He, Long Yun, Xiang Zhou, Xu Wang, Yi Chi, Siyi Yuan, Zhanqi Zhao

**Affiliations:** 1 Department of Critical Care Medicine, Peking Union Medical College Hospital, Peking Union Medical College, Chinese Academy of Medical Sciences, 1 Shuaifuyuan, Dongcheng District, Beijing, China; 2 Department of Biomedical Engineering, Fourth Military Medical University, Xi’an, China; 3 Institute of Technical Medicine, Furtwangen University, Villingen-Schwenningen, Germany

**Keywords:** High-flow nasal cannula, Electrical impedance tomography, Lung recruitment, Lung overdistension

## Abstract

**Background:**

Postextubation high-flow nasal cannula (HFNC) is used as a support therapy in high-risk patients in ICU. This study aimed to determine the effects of HFNC therapy on lung recruitment and overdistension assessed by electrical impedance tomography (EIT).

**Methods:**

Twenty-four patients who received HFNC within 24 h after extubation were prospectively enrolled in this study. EIT was used to monitor regional lung ventilation distributions at baseline (conventional oxygen therapy) and three flow rate levels of HFNC therapy (20, 40, and 60 L/min). Change of end-expiratory lung impedance (ΔEELI), regional recruitment (recruited-pixels) and overdistension (overdistended-pixels), and lung strain change were determined by EIT. EIT images were equally divided into four ventral-to-dorsal horizontal regions of interest (ROIs 1, 2, 3, and 4). “Overdistension-_by HFNC_” due to HFNC is defined as an increase of overdistened-pixels > 10 than baseline. Patients were divided into two groups: (1) high potential of recruitment (HPR), recruited-pixels > 10 pixels at 60 L/min than baseline, and (2) low potential of recruitment (LPR), recruited-pixels < 10 pixels at 60 L/min than baseline.

**Results:**

When the flow rate gradually increased from baseline to 60 L/min, a significant and consistent increasing trend of global ΔEELI (%) (*p* < 0.0001), recruited-pixels (*p* < 0.001), and overdistended-pixels (*p* = 0.101) was observed. Moreover, the increase of ΔEELI was mainly distributed in ROI2 (*p* = 0.001) and ROI3 (*p* < 0.0001). The HPR group (13/24 patients) had significantly higher recruited-pixels than the LPR group (11/24 patients) at 20, 40, and 60 L/min. There were no significant differences in PaO_2_/FiO_2_, ΔEELI (%), and overdistention pixels between the two groups. The HPR group had 13 patients in which no one had “overdistension-_by HFNC_”, and the LPR group had 11 patients in which 4 patients had “overdistension-_by HFNC_” (0/13 vs. 4/11, *p* = 0.017).

**Conclusions:**

Using EIT could identify diverse effects of HFNC on lung regional ventilation in postextubation situations. Further study is required to validate using “HFNC effect” based on lung recruitment and overdistension by EIT in clinical practice.

**Trial registration:**

The study was retrospectively registered at www.clinicaltrials.gov (no. NCT04245241).

## Key messages

- Using EIT could identify the effects of HFNC on lung regional recruitment and overdistension. Moreover, the effect varied among individuals.
- Classification of “HFNC effect” based on lung recruitment/non-recruitment and overdistension/non-overdistension might be helpful to guide HFNC therapy in post-extubation situations. Further study will be required to validate the meaning of this classification in clinical practice.

## Background

The utilization of high-flow nasal cannula (HFNC) therapy in the ICU has gained great attention as a bridge therapy after early extubation. It has been used as a first-line strategy for extubation patients at a high risk of hypoxemia [1, 2]. Recent studies have shown that HFNC therapy improves respiratory drive and lung mechanics [3] and enhances CO_2_ removal [4–6]. A number of studies have described low levels of airway pressure associated with increased end-expiratory lung volume and improved oxygenation with HFNC therapy [4, 7–13]. Moreover, HFNC can improve global and regional lung aeration [3, 14]. In all these clinical and physiologic studies, the set HFNC flow rates were extremely heterogeneous, ranging between 15 and 100 L/min [1, 8, 11, 15]. This may be related to the complexity of the estimated gas distribution of different flow rates and may make it difficult to select an appropriate flow rate for individual patients.

However, Piastra et al. reported uncommon barotrauma using HFNC therapy (2 L/kg/min, FiO_2_ 0.6) in young infants [16]. Thus, the lung injury induced by HFNC therapy should also be carefully considered. Lung overdistension and stress and strain are the primary determinants of ventilator-induced lung injury [17]. Electrical impedance tomography (EIT) is a noninvasive, non-ionizing bedside method of monitoring impedance changes related to different lung conditions, especially those related to regional ventilation, such as lung regional recruitment and overdistension during PEEP titration in ARDS patients [18, 19]. To our knowledge, no one has quantified lung regional response (recruitment, overdistension, and cyclic alveolar collapse) to HENC therapy at different flow levels in critically ill patients.

The aims of this study were as follows: (1) to investigate the effects of HFNC on lung recruitment and overdistension by EIT and (2) to explore a potential method of using EIT for optimal HFNC therapy in clinical practice.

## Patients and methods

### Patients

The Institutional Research and Ethics Committee of the Peking Union Medical College Hospital approved this study for human subjects. Written informed consent was obtained from all patients or next of kin before data were included in the study.

When the research team was available, adult critically ill patients who received HFNC therapy within 24 h after the extubation were enrolled. Local indications of HFNC therapy after extubation were the following: (1) high-risk of acute respiratory failure after extubation, such as major surgery, basic cardiac dysfunction, basic lung dysfunction, and mechanical ventilation > 7 days, and (2) presentation of occult respiratory failure after extubation—PaO_2_/FiO_2_ < 300, SpO_2_ < 92%, and/or respiration rate > 25 bpm. Patients were excluded from the study if they were aged < 18 years, were pregnant, had a body mass index (BMI) over 50 kg/m^2^, had ribcage malformation, or had any contraindication against using EIT monitoring (automatic implantable cardioverter defibrillator, chest skin injury, etc.).

### Physiological measurements

At enrollment, we collected physiological data at baseline, including height and weight, to assess the BMI, APACHE-II score, days of intubation, and PaO_2_/FiO_2_ ratio. Respiratory (RR), peripheral oxygen saturation (SpO_2_), ROX index (ratio of SpO_2_/FiO_2_ to RR), and hemodynamic parameters, including heart rate (HR) and mean arterial pressure (MAP), were obtained at different flow rates. Moreover, the inspiratory and expiratory times were derived from the EIT curve, and PI was measured via the patient’s finger by using the IntelliVue MP70 monitor (Philips Medical Systems, Boblingen, Germany). The MP70 system calculates the PI as the ratio between the pulsatile component and the non-pulsatile component of the light reaching the light-sensitive cell of the pulse oximetry probe.

### Experimental protocol

The flow rate of HFNC was gradually increased to achieve the preset three levels (20, 40, and 60 L/min), and each flow level was maintained for 20 min to maintain a balance of airway pressure. The entire study lasted approximately 80 min. The patient’s condition before HFNC therapy was defined as baseline, which also was taken as zero flow rate of HFNC. Moreover, patients were in a semi-recumbent position during the study period. Conditioned medical air (FiO_2_ 0.30) was delivered via an HFNC (Optiflow, Fisher & Paykel Healthcare, Auckland, New Zealand). The humidifier (MR850, Fisher & Paykel Healthcare, Auckland, New Zealand) temperature was set to 37 °C, and the air was delivered by medium-sized, silicon nasal cannulae (RT050/051, Fisher & Paykel Healthcare, Auckland, New Zealand). The subjects were asked to breathe with their mouth closed during the procedure.

### EIT measurements

EIT measurements were obtained with PulmoVista (Dräger Medical, Lübeck, Germany). During the protocol, a silicone EIT belt with 16 surface electrodes was placed around the patient’s thorax in one transverse plane corresponding to the 4th intercostal parasternal space and was then connected to the EIT monitor for bedside visualization. Electrical alternating currents were applied in a sequential rotating process through adjacent electrode pairs. The resulting differences in surface potential between neighboring electrode pairs were measured. The stimulation frequency and amplitude were adjusted automatically by the EIT device to minimize the influence of background noise. EIT measurements were continuously performed at 20 Hz. In addition, the data were digitally filtered using a low-pass filter with a cutoff frequency of 0.67 Hz to eliminate cardiac-related impedance changes. EIT scans consist of images showing impedance with a 32 × 32-color-coded matrix. EIT data were analyzed offline according to a previously described EIT-based algorithm [20].

### Analysis and definition of EIT data

To reduce the heterogeneity of spontaneous breathing, 2 min of continuous EIT data (with representative breaths) was selected for data analysis (data were averaged) at each flow rate [3, 7].

Moreover, the change of EELI (ΔEELI) and the change of TV (ΔTV) were determined relative to the baseline EELI and TV. The related definitions were the following:

- EIT images were divided into four symmetrical, non-overlapping and ventral-to-dorsal horizontal regions of interest (ROIs), ranging from the gravity-independent areas to the gravity-dependent areas, namely, the ventral (ROI1), mid-ventral (ROI2), mid-dorsal (ROI3), and dorsal (ROI4) regions. Gravity-independent regions were defined as ROI1 + ROI2, and gravity-dependent regions were defined as ROI3 + ROI4.
- EIT images at end-inspiration and end-expiration were identified. Tidal image = end-inspiration image − end-expiration image [20].
- End-expiration image pixels = all pixels higher than 25% of the maximum pixel value in the image. Tidal image pixels = all pixels higher than 20% of the maximum value in the image of tidal breathing [18].
- Recruited regions (pixels) are defined as newly aerated pixels at different flow rates of 20, 40, and 60 L/min compared to baseline [20].
- Overdistended regions (pixels) are defined as aerated pixels that did not join in tidal ventilation at the same flow rate [20]. Hence, the overdistended region pixels are equal to “end-expiration image pixels” minus “tidal image pixels”.
- Δoverdistended-pixels = Difference in overdistended pixels between 20/40/60 L/min than baseline; moreover, “overdistension-_by HFNC_” caused by HFNC was defined as an increase of Δoverdistended-pixels > 10 at 60 L/min than baseline.
- Tidal recruitment/derecruitment (cyclic alveolar collapse) regions (pixels) are defined by new aerated pixels from tidal variation to end expiration [20].
- Patients were divided into two groups: (1) high potential of recruitment (HPR), recruited-pixels > 10 pixels at 60 L/min than baseline, and (2) low potential of recruitment (LPR), recruited-pixels < 10 pixels at 60 L/min than baseline.

### Statistical analysis

Statistical analyses were performed using Prism 7 (GraphPad Software, San Diego, CA, USA) and the SPSS 24.0 software package (SPSS Inc., Chicago, IL, USA). This is a self-controlled paired study, and the sample size was calculated using the following formula: *n* = (Z1 − *a*/2 + Z1 − *β*)^2^Sd^2^/(μt − μc)^2^

where *α* is 0.05, *β* is 0.1, and Sd is the standard deviation of the difference before and after the HENC intervention. μt − μc is the difference between the follow-up and baseline groups. The sample size was estimated based on ΔEELI (%) [3]. The mt (SD) and mc (SD) were 74 (174) and 230 (237) based on a previous study, respectively, with a correlation coefficient of 0.6. The minimum sample size to obtain a study power of 90% was 18 pairs. The Mann–Whitney test was used for comparisons of groups (HPR group vs. LPR group). Normal distribution was assessed with Kolmogorov-Smirnov normality test. Normally distributed results are presented as mean ± SD whereas non-normally distributed results are presented as median (25th–75th percentile). Comparisons of the trends of the related parameters according to the different flow rates were performed using a General Linear Model Repeated Measures (GLMRM) [21, 22]. This model is an extension of the classical ANOVA, which allows handling both fixed effect (different flow) and random effect (patient). GLMRM takes into account the correlation between multiple measurements on one patient and thus the estimated marginal means were adjusted for the covariates and the trends of related EIT parameters corresponding to the different flow. When Mauchly’s test of sphericity is not demanded (*p* < 0.05), Epsilon (Greenhouse-Geisser) was used for the corrected test [23]. All statistics were two-tailed, and a *p* value of less than 0.05 was considered to be significant.

## Results

From May 2018 to July 2019, a total of 24 patients were enrolled. The main characteristics of the patients are summarized in Table 1. The mean patient age was 66 ± 11, and 10/24 (53%) were men. Moreover, the mean days of mechanical ventilation before extubation was 5 ± 3. Twenty-two postoperative patients were intubated and mechanically ventilated at the beginning of anesthesia. The other two patients received invasive ventilation because of respiration failure.

**Table 1:** Main characteristics of the study population

Pts	Age	F/M	BMI	APACHE-II	Admission category	Days of intubation	PaO_2_/FiO_2_	PaCO_2_ (mmHg)
1	63	M	27	11	Thoracic operation	7	375	36
2	63	F	28	13	Abdominal operation	4	375	36
3	77	F	22	11	Thoracic operation	4	291	35
4	67	M	27	8	Cardiosurgery	4	235	36
5	68	M	17	15	Cardiosurgery	9	181	36
6	53	M	24	7	Cardiosurgery	9	269	36
7	82	M	24	15	Cardiosurgery	7	323	36
8	81	F	24	7	Abdominal operation	3	303	47
9	67	M	28	16	Abdominal operation	3	308	36
10	32	M	18	6	Thoracic operation	2	184	42
11	80	F	18	15	Cardiosurgery	7	275	50
12	73	M	28	21	Abdominal operation	7	417	53
13	63	F	25	11	Abdominal operation	5	325	30
14	67	M	22	9	Abdominal operation	5	295	50
15	63	F	22	9	Abdominal operation	1	161	35
16	65	M	19	12	Thoracic operation	10	275	38
17	68	F	25	10	Thoracic operation	10	182	39
18	65	F	23	15	Abdominal operation	3	155	38
19	65	F	23	12	Other	4	220	37
20	66	M	22	14	Abdominal operation	5	271	38
21	68	M	24	16	Abdominal operation	6	284	40
22	66	M	23	12	Thoracic operation	5	273	41
23	64	M	20	10	Cardiosurgery	4	264	39
24	66	M	26	14	Other	5	276	43
Summary	66 ± 11	9/15	23 ± 3	12 ± 4	22/24 postoperative pts	5 ± 3	266 ± 69	40 ± 6

*BMI* body mass index (kg/m^2^), *APACHE-II* Acute Physiology and Chronic Health Evaluation II, *FiO*_*2*_*(%)* fraction of inspired O_2_, *M* male, *F* female, *pts* patients

### Effects of HFNC therapy on ΔEELI and lung recruitment, overdistension, and tidal recruitment at different flow rates

**Fig. 1:**
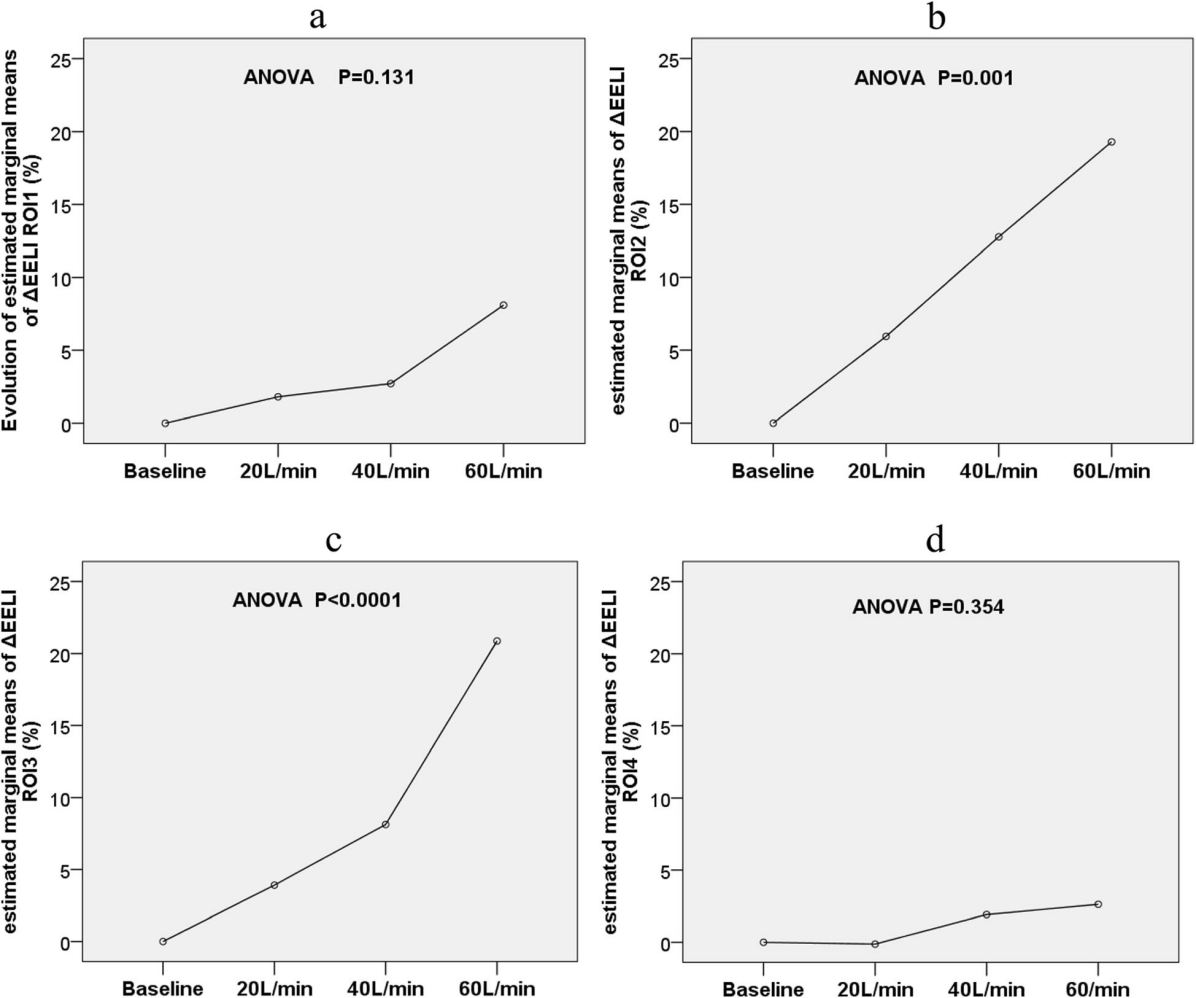
Evolution of estimated marginal means of ΔEELI ROI1 (%), ΔEELI ROI2 (%), ΔEELI ROI3 (%), and ΔEELI ROI4 (%) at different flow rates

The trend of global ΔEELI significantly increased (*p <* 0.0001). The increased gas volume was mainly distributed in ROI2 (*p* = 0.001) and ROI3 (*p* < 0.0001), but the trend in ROI1 (*p* = 0.131) and ROI4 (*p* = 0.345) was not significant from baseline to 60 L/min flow rate (Fig. 1).

The recruitment regions at flow rates of 20, 40, and 60 L/min were 5 (5,17), 9 (1,27), and 11 (1,22) pixels (*p* < 0.0001), respectively. Moreover, the global and regional ΔTV, overdistended pixels, and tidal recruitment /derecruitment pixels did not change significantly with the introduction of HFNC therapy compared to baseline (Table 2).

**Table 2:** Evolution of EIT-related parameters at different flow rates

Variables	Baseline	20 L/min	40 L/min	60 L/min	Trend—*p*	Mauchly‘s test of sphericity *p* value
ΔEELIgl (%)	Baseline	7.19 (− 5.12,18.66)	10.61 (− 3.42,41.99)^a,b^	26.32 (10.68,60.75)^a,b,c^	< 0.0001*	0.046
ΔEELI ROI1 (%)	Baseline	0.32 (− 0.07,2.74)	0.18 (0.00,3.05)	1.61 (0.00,4.60)	0.131	0.000
ΔEELI ROI2 (%)	Baseline	2.12 (− 3.21,7.35)	8.6 (− 1.42,18.19)^a,b^	16.15 (4.00,27.90)^a,b^	0.001*	0.000
ΔEELI ROI3 (%)	Baseline	2.52 (− 3.77,6.23)	3.94 (− 3.04,14.48)	14.29 (4.01, 32.26)^a,b,c^	< 0.0001*	0.462
ΔEELI ROI4 (%)	Baseline	0.21 (0.00,0.40)	0.00 (0.00,1.77)	0.33 (0.00,2.43)	0.345	0.000
ΔVTgl (%)	Baseline	6.40 (− 12.27,22.74)	5.66 (− 8.60,19.78)	3.71 (− 3.29,15.29)	0.095	0.066
ΔVT ROI1 (%)	Baseline	0.41 (− 1.13,1.72)	0.43 (− 0.27,2.10)	0.72 (0.00,2.53)	0.069	0.000
ΔVT ROI2 (%)	Baseline	1.66 (− 5.01,13.19)	4.43 (− 2.17,12.41)	1.93 (− 2.50,13.52)	0.077	0.003
ΔVT ROI3 (%)	Baseline	2.97 (− 4.07,8.10)	0.53 (− 4.20,8.00)	0.12 (− 9.02,10.20)	0.446	0.004
ΔVT ROI4 (%)	Baseline	0.00 (− 1.19,0.64)	0.00 (− 0.80,1.23)	0.00 (− 0.54,1.24)	0.354	0.000
GI index (%)	0.47 ± 0.22	0.47 ± 0.21	0.47 ± 0.23	0.46 ± 0.20	0.693	0.000
RVD index (%)	4.34 (3.39,9.65)	5.66 (3.74,11.82)	5.52 (3.51,9.59)	5.89 (3.82,8.34)	0.191	0.000
Recruited pixels	Baseline	5 (5,17)^a,b^	9 (1,27)^a,b^	11 (1,22)^a,b^	0.0001*	0.000
Overdistended pixels	14 (8,30)	13 (5,28)	13 (3,31)	15 (6,32)	0.101	0.000
Tidal recruitment/derecruitment pixels	12 (7,41)	9 (1,16)	16 (8,33)	11 (4,16)	0.124	0.031

*p* value by one-way analysis of variance (ANOVA) for repeated measures

*ΔEELI* change of end-expiratory lung impedance, *ΔVT* change of tidal variation, *gl* global, *ROI* region of interest, *GI* global inhomogeneity, *RVD* regional ventilation delay

^a^vs. baseline, *p* < 0.05

^b^vs. 20 L/min, *p* < 0.05

^c^vs. 40 L/min, *p* < 0.05

*p < 0.05

### Effects of HFNC therapy on respiratory and hemodynamic parameters

Regarding hemodynamic parameters, no significant changes were observed during the study phases (Table 3). Moreover, inspiration (*p* = 0.345) and expiration times (*p* = 0.967), ROX index (*p* = 0.120), and RR (*p* = 0.144) remained stable over all study phases. However, a significant change trend of SpO_2_ (*p* = 0.029) was found during the various flow rate change.

**Table 3:** Change of respiratory and hemodynamics parameters at different flow rates

Variables	0 L/min	20 L/min	40 L/min	60 L/min	Trend–*p*	Mauchly’s test of sphericity *p* value
SPO_2_ (%)	97 ± 2	98 ± 2	98 ± 2	98 ± 2	0.029*	0.326
RR (bpm)	22 ± 6	20 ± 4	20 ± 3	21 ± 5	0.144	0.060
ROX index	22.41 ± 5.38	23.88 ± 5.20	23.54 ± 4.35	22.20 ± 4.34	0.120	0.598
Inspiration time(s)	1.48 ± 0.34	1.61 ± 0.56	1.54 ± 0.40	1.55 ± 0.42	0.345	0.891
Expiration time (s)	1.72 ± 0.59	1.71 ± 0.48	1.72 ± 0.46	1.78 ± 0.65	0.967	0.755
MAP (mmHg)	86 ± 10	86 ± 10	87 ± 8	87 ± 8	0.358	0.147
HR (bpm)	90 ± 14	89 ± 13	89 ± 14	88 ± 13	0.338	0.000
PI	1.9 ± 1.2	2.0 ± 1.2	1.6 ± 1.0	1.9 ± 1.1	0.635	0.000

*SPO*_*2*_ peripheral oxygen saturation, *RR* respiratory rate, *HR* heart rate, *MAP* mean arterial pressure, *PI* peripheral perfusion index

ROX index = (Respiratory rate-OXygenation) index = the ratio of SpO_2_/FiO_2_ to RR

*p* value by one-way analysis of variance (ANOVA) for repeated measures

*p < 0.05

### Differences between HPR and LPR groups

Thirteen patients could be categorized to the HPR group (in which 12 patients were postoperative), and 11 patients to the LPR group (in which 10 patients was postoperative). Baseline characteristics of HPR and LPR groups are compared in Table 4. There were no significant differences in PaO_2_/FiO_2_, ROX index, and days of intubation between these two groups at baseline.

**Table 4:** Comparison baseline date in high potential of recruitment (HPR) and low potential of recruitment (LPR) groups

Variables	HPR group, *N* = 13	LPR group, *N* = 11	*p* value
Age	65 ± 12	68 ± 5	1.000
F/M	4F/9M	6F/5M	0.239
BMI	23 ± 4	24 ± 2	0.910
APACHE-II	12 ± 3	12 ± 4	0.649
Days of intubation	6 ± 4	6 ± 2	1.000
PaO_2_/FiO_2_	284 ± 74	257 ± 59	0.331
PaCO_2_ (mmHg)	40 ± 7	39 ± 4	0.955

*BMI* body mass index (kg/m^2^), *APACHE-II* Acute Physiology and Chronic Health Evaluation II, *FiO*_*2*_*(%)* fraction of inspired O_2_, *M* male, *F* female

**Fig. 2:**
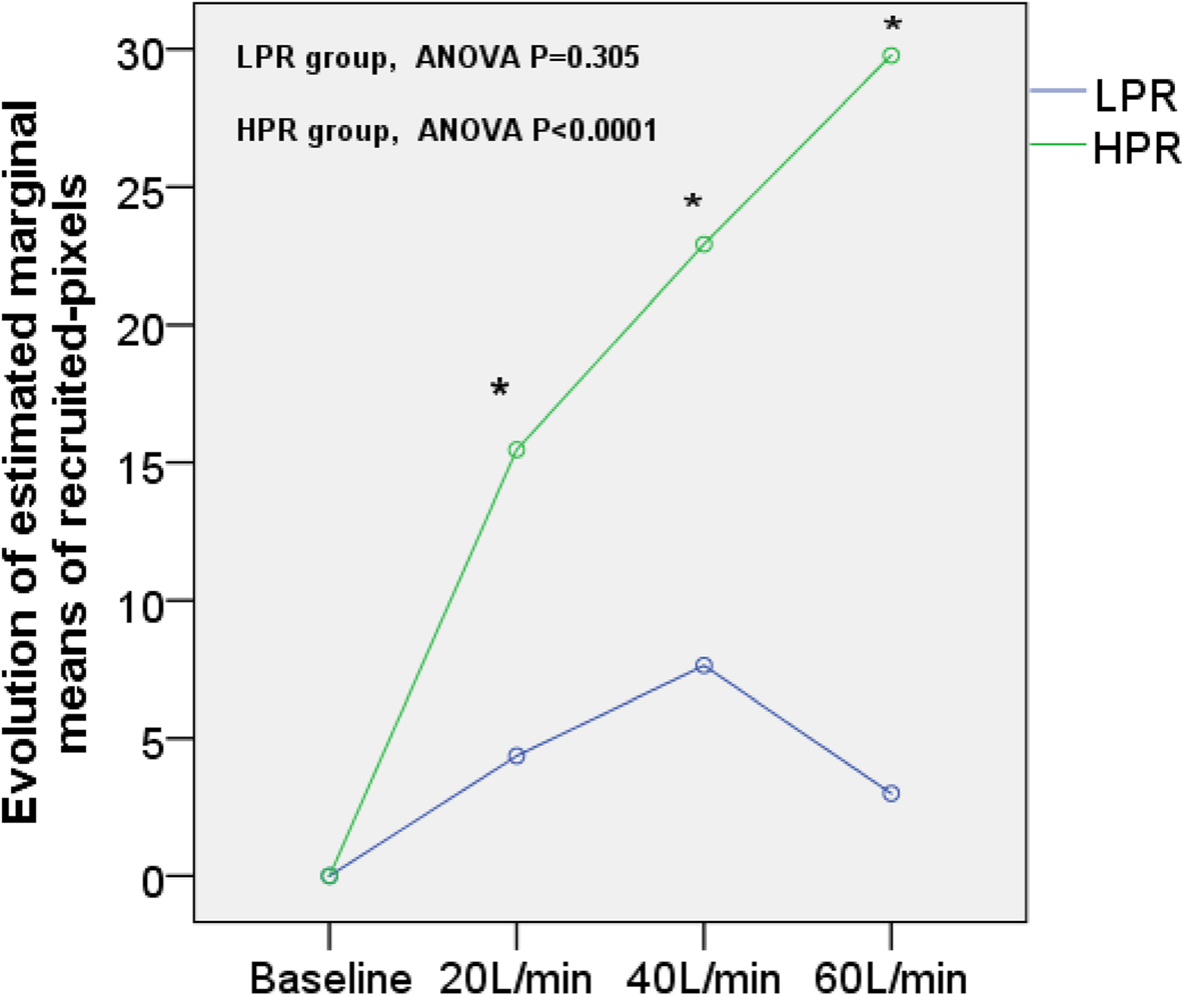
Evolution of estimated marginal means of recruited-pixels at different flow rates between the HPR and LPR groups. *p < 0.05, HPR vs. LPR at the same flow rate

Evolutions of related parameters between HPR and LPR groups at different flows are shown in Table 5. The estimated marginal means of recruited-pixels at different flow rates between HPR and LPR groups are shown in Fig. 2. The HPR group had a significant higher recruited regions (pixels) than the LPR group at different flow rates (20 L/min, 12 (7,24) vs. 0 (0,5) *p* = 0.001; 40 L/min, 17 (10,41) vs. 0 (0,8) *p* = 0.002; and 60 L/min, 20 (16,42) vs. 0 (0,8) *p* < 0.0001). There was no significance in overdistension-pixels, tidal recruitment/derecruitment (pixels), and Δoverdistension-pixels (from baseline) between the two groups. However, the LPR group had a significantly higher percentage of “overdistension-_by HFNC_” patients than the HPR group (0/13 vs. 4/11, *p* = 0.017).

**Table 5:** Change parameter of High potential of recruitment (HPR) group vs Low potential of recruitment (LPR) group at different follow

Variables	Baseline	20 L/min	40 L/min	60 L/min	Trend—*p*	Mauchly’s test of sphericity *p* value
ΔEELIgl (%)						
HPR group	Baseline	7.03 (− 4.49,21.30)	30.66 (0.33,47.35)^a,b^	32.31 (10.51,102.96)^a,b^	0.007	< 0.0001
LPR group	Baseline	7.46 (− 7.67,17.80)	5.56 (− 9.20,28.96)	43.95 (11.53,71.69)	0.003	0.754
ΔVTgl (%)						
HPR group	Baseline	9.98 (− 1.13,21.14)	11.62 (− 8.21,20.99)	4.27 (− 6.75,16.14)	0.194	0.023
LPR group	Baseline	4.80 (− 25.12,41.99)	1.64 (− 9.57,20.06)	0.12 (− 3.42,38.45)	0.305	0.242
Recruitment region (pixels)						
HPR group	Baseline	12 (7,24)^a,¶^	17 (10,41)^a,b,¶^	20 (16,42)^a,b,c,¶^	< 0.0001	0.013
LPR group	Baseline	0 (0,5)	0 (0,8)	0 (0,8)	0.305	0.242
Overdistension (pixels)						
HPR group	10 (5,31)	10 (5,30)	11 (1,33)	13 (3,33)	0.456	0.003
LPR group	16 (9,22)	18 (6,27)	22 (4,28)	16 (8,26)	0.159	0.006
Δoverdistension (pixels)						
HPR group	Baseline	1 (− 2,4)	1 (− 1,6)	3 (1,9)	0.456	0.003
LPR group	Baseline	1 (− 4,8)	1 (− 4 ,10)	4 (− 1,7)	0.159	0.006
Tidal recruitment/derecruitment (pixels)						
HPR group	15 (4,38)	8 (0,15)	15 (1,33)	5 (2,18)	0.096	0.77
LPR group	11 (7,51)	10 (7,27)	19 (10,33)	12 (6,13)	0.572	0.08
RR (bpm)						
HPR group	21 (19,25)	20 ± 3	20 ± 3	21 ± 4	0.029	0.390
LPR group	20 (17,25)	21 ± 5	20 ± 3	23 ± 5	0.473	0.270
ROX index						
HPR group	22 ± 4	24 ± 4	23 ± 4	23 ± 5	0.065	0.089
LPR group	23 ± 7	23 (19,28)	24 ± 5	21 ± 4	0.252	0.664
SPO_2_ (%)						
HPR group	97 ± 2	97 ± 2	98 ± 2	97 ± 2	0.211	0.259
LPR group	97 (95,100)	98 ± 2	99 (98,100)	98 (97,100)	0.197	0.171

^a^vs. baseline, *p* < 0.05;

^b^vs. 20 L/min, *p* < 0.05;

^c^vs. 40 L/min, *p* < 0.05;

^¶^*p* < 0.05 for HPR vs. LPR at the same flow

## Discussion

In extubated patients within 24 h of undergoing HFNC therapy, in addition to improving the EELI and lung aeration distribution, a high flow rate also induces regional recruitment and overdistension. However, those effects varied among individuals.

### Effects of HFNC therapy on lung recruitment, overdistension, and tidal recruitment

As shown in a previous study, HFNC may induce lung injury barotraumas in infants [16]. In our study, we found that HFNC therapy may induce overdistension, which may be related to the lung injury. Additionally, compared to baseline, HFNC therapy could decrease pulmonary atelectasis and increase overdistension. Meanwhile, tidal recruitment/derecruitment (also present in healthy lungs [24]), which reflects the potential of lung recruitment, was not decreased significantly by HFNC therapy. Furthermore, we distributed all patients into two types from 0 L/min to 60 L/min: (a) in HPR, thirteen patients showed recruitment (mainly in the dorsal region) with decreased tidal recruitment/derecruitment and without lung overdistension and (b) in LPR, seven patients showed neither recruitment nor overdistension and four patients showed overdistension (mainly in the ventral region) without lung recruitment. Thus, patients classified as type HPR may have a greater potential for recruitment and a lower risk of overdistension, which indicates that HFNC therapy may benefit these patients and that higher flow rates may be required. Patients classified as type LPR may have limited potential for recruitment and a higher risk of hyperinflation, which indicates that HFNC therapy may induce lung injury and should be used cautiously and that the flow rate should be reduced. Moreover, higher flow rates may induce lung recruitment and may lead to hyperinflation in small patients of type LPR, which requires further research. Therefore, HFNC therapy is likely to be beneficial to most patients but can induce lung injury in a small proportion of patients; thus, the flow rate should be selected cautiously among different patients.

### Effects of HFNC therapy on lung strain change

High values of dynamic lung strain (lung deformation caused by tidal volume) and static lung strain (lung deformation caused by positive end-expiratory pressure (PEEP)) are associated with lung injury [25–27]. However, little is known regarding the influence of flow rates on lung strain during HFNC therapy. EIT has been used to evaluate the lung regional effects of HFNC on change of global and regional end-expiratory lung impedance (EELI) [20, 28], along with change of tidal variation (ΔTV) [29]. Protti et al. showed that in an animal model, for the same global strain, a large static strain is less harmful than a large dynamic strain [19]. In the present study, lung strain could also be deduced by the related EIT parameters (deduction procedure was documented in the online Additional file 1). Given a stable unchanged tidal volume, patients classified as HPR (type HPR, with lung recruitment while receiving HFNC therapy) may have lower dynamic and static lung strain, and patients classified as LPR (types LPR, without lung recruitment while receiving HFNC therapy) have unchanged dynamic lung strain and higher static lung strain, meaning that these patients may have a higher risk of lung injury. Therefore, HFNC therapy may be beneficial for some patients (HPR) by inducing lung recruitment without causing overdistension and may be harmful for patients (LPR) who do not show lung recruitment while receiving HFNC therapy, which may be a foundation for daily clinical flow rate selection.

### Effects of HFNC therapy on lung aeration distribution

Previous studies showed that HFNC may increase the lung volume (EELI) [3, 14]; we also found the same result. Furthermore, we also found that early extubated patients exhibited heterogeneity in the gas distribution between the gravity-independent and gravity-dependent lung regions because of atelectasis in the gravity-dependent region. In addition, as the flow rate increased, the EELI of the gravity-dependent region also increased due to recruitment. Moreover, the high flow reopened the medial-ventral and medial-dorsal regions of the lung, showing heterogeneity, mainly because of the limited airway pressure of HFNC therapy (Parke et al. observed that for each increase of 10 L/min in the flow rate, the mean airway pressure increased by 0.69 cm H_2_O [24]). Thus, it is necessary to monitor the gas distribution to avoid ventilation heterogeneity and to select an appropriate flow rate when using HFNC therapy.

### Effects of HFNC therapy on hemodynamics and respiratory parameters

Regarding hemodynamics, we did not find obvious differences among the flow rates, meaning that 20 min of HFNC therapy may have little influence on hemodynamics. Moreover, several studies conducted in patients with high baseline RR (25 ± 3 breaths/min) [4, 9] have shown that HFNC therapy may improve the breathing pattern. In addition, the ROX index also was used to assess the effect of HFNC therapy and predict the outcome of HFNC [30–32]. However, we did not find such an effect (decrease of RR and increase of ROX index) of HFNC in the present study. Several potential reasons are the following: (a) Most of the patients were at relatively normal respiratory status without obvious hypoxia (SpO_2_ 97 ± 2%, RR 22 ± 6 bpm) at the baseline. In the present study, the primary aim of using high-flow nasal cannula therapy to prevent respiratory failure in a high-risk patient; (b) the inability of the RR (approximately 20 breaths/min) to be changed in such a short time period (20 min).

### Study strengths and limitations

**Fig. 3:**
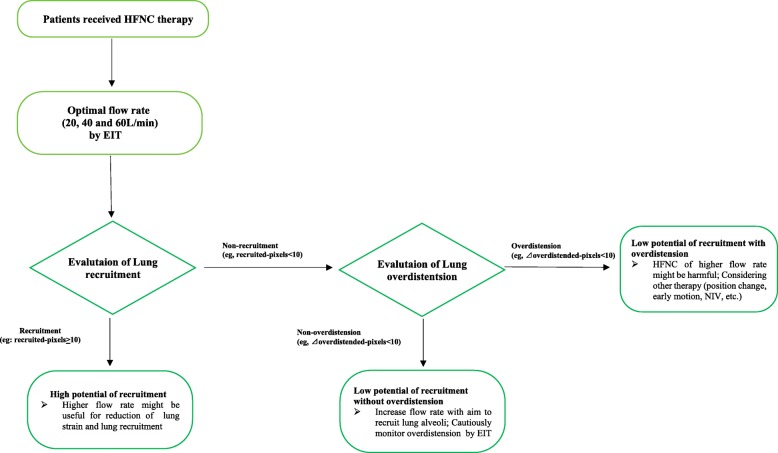
Conceptual schematic for using EIT to guide HFNC therapy. NIV, non-invasive ventilation

Our study needs to be interpreted within the context of its strengths. The greatest interest of this study is that a heterogeneous response to different HFNC flows was found in this study. Marui et al. reported that an increasing HFNC flow rate progressively decreased inspiratory effort and improved lung aeration, dynamic compliance, and oxygenation in patients with acute hypoxemic respiratory failure [3]. However, some contrary results were reported in different studies using HFNC for post-extubation patients. Corley et al. found that HFNC did not improve atelectasis, oxygenation, respiratory rate, or dyspnea, nor did it reduce rates of failure of allocated therapy in recently extubated obese patients [33]. Maggiore et al. showed that HFNC improved oxygenation, comfort, and the reintubation rate in the post-extubation setting [8]. Our study also supported the idea that HFNC should be of benefit to some selected patients rather than to all patients [34]. In the present study, different patients had different responses to HFNC: 13 patients showed recruitment without overdistension, 7 patients showed neither recruitment nor overdistension, and 4 patients showed non-recruitment with overdistension. These results may be helpful for clinicians to select appropriate flow rates. However, how exactly to use the EIT approach to (1) decide if a patient should receive HFNC and (2) titrate an optimal flow rate for those who need HFNC will require further research. Based on the implications of these findings, a conceptual schematic for using EIT guide HFNC therapy is presented in Fig. 3.

Several limitations should be acknowledged. First, the study period may be considered to be not long enough to evaluate other relevant clinical outcomes. Second, EIT images display approximately one-third the area of the lungs; therefore, they cannot be used to measure lung volume changes along the vertical axis. Nevertheless, previous studies have shown good agreement between the findings of EIT and those of other reference methods that measure the whole lung volume [35]. Third, while HFNC therapy may induce both lung recruitment and overdistension in some patients at the same time, this phenomenon was not reproduced in our study because the pressure produced by HFNC therapy was large enough. However, for the safety of the patients, we did not choose flow rates higher than 60 L/min. Fourth, our study compared different flow rates in an effort to assess an optimal flow rate (induce recruitment with minimal overdistension), but the data were not conclusive; thus, further study is required. Moreover, using recruited-pixels > 10 as regional lung recruitment and overdistended-pixels > 10 as regional overdistension might be arbitrary; thus, further study is required to validate the related cutoff values. However, we showed lung recruitment, overdistension, and tidal recruitment/derecruitment at baseline and at different flow rates, and HFNC for 20 min was able to decrease the dynamic and static lung strain in some patients. Fifth, working of breathing (WOB) and diaphragm protection were not measured in the present study. Reduction of WOB and diaphragm protection might be more relevant than lung recruitment during the HFNC therapy in postoperative patients. Further study is required to investigate the relationship of diaphragm protection and lung recruitment during the HFNC therapy. Combining diaphragm protection and lung recruitment might be more suitable and effective. Sixth, air trapping may exist in smokers, which may affect the overdistension results; however, we did not detect the phenomenon in this study. Seventh, with the aim to titrate an optimal flow rate in practical terms, the three phases of different flow rates were stepwise elevated but not be randomized. To control the carryout effect, the flow rate gradually increased (from 0 → 20 → 40 → 60 L/min). An increased flow rate was accompanied by the elevation of airway pressure, and theoretically, the former (low) flow rate had little effect on the later (high) flow rates. However, 20 min in each period should have been sufficient to achieve a stable effect on lung volume and gas exchange in this study. Parke et al. reported that airway pressure can stabilize in 15 min during HFNC therapy [36]. Eighth, some might argue regarding the difference of air trapping and lung overdistension by EIT. Overinflated regions of lungs might sometimes appear similar to air trapping. Air trapping is defined as lung overdistension due to flow limitation in COPD/asthma/ARDS [37, 38]. Two cases that reported using EIT technology could be useful to adjust an individual extra-PEEP of mechanical ventilation settings in severe asthma patients [38, 39]. In the present study, we did not find any signs of air trapping by EIT (flow limitation) as the flow rate increased. Hence, the potential effect of air trapping may not impact our conclusions.

## Conclusions

There were diverse responses of lung regional ventilation and aeration (recruitment, non-recruitment, and overdistension) to HFNC. However, the effect varied among individuals. Identification of lung recruitment, lung non-recruitment, and overdistension by EIT might be helpful to guide HFNC therapy in post-extubation situations. Further study will be required to validate the meaning of this classification in clinical practice.

## Supplementary information

**Additional file 1.** Deduction procedure and calculation formula of lung strain.

## Data Availability

The datasets used and/or analyzed during the current study are available from the corresponding author on reasonable request.

## Abbreviations

ANOVA: Analysis of variance
BMI: Body mass index
EIT: Electrical impedance tomography
FiO_2_: Fraction of inspired oxygen
GI: Global inhomogeneity
HFNC: High-flow nasal cannula
HR: Heart rate
MAP: Mean arterial pressure
PI: Perfusion index
ROIs: Regions of interest
RR: Respiratory rate
RVD: Regional ventilation delay
SPO_2_: Peripheral oxygen saturation
ΔEELI: Change of end-expiratory lung impedance
ΔTV: Change of tidal variation
