# Supplementary material for: Effect of postextubation high-flow nasal cannula therapy on lung recruitment and overdistension in high-risk patient
Source: Crit Care. 2020 Mar 6;24:82. doi: 10.1186/s13054-020-2809-7 (PMC7060646; doi:10.1186/s13054-020-2809-7)
Supplement: Supplementary file 1 — Additional file 1. Deduction procedure and calculation formula of lung strain. [file 13054_2020_2809_MOESM1_ESM.docx]

**E****f****fect of** **postextubation high-flow nasal cannula therapy on lung recruitment and overdistension in high-risk patient**

**Changes of lung strain and deduction procedure**

**1.Definition and calculation of lung strain parameters**

The global lung strain consists of dynamic and static lung strain [1, 2]. Dynamic lung strain is defined as the ratio between tidal volume and functional residual capacity (FRC) at the baseline [3]. Static lung strain is defined as the ratio between the in-crease of aeration caused by an increase of airway pressure and the FRC [4]. EIT technology have been proven to effectively measure the change of tidal volume and end-expiratory lung volume by impedance monitoring [5-8]. Hence, EIT-derived parameters were used to assess the lung strain in the present study.

The related definitions were the following:

● ΔIrecruit, is defined as the increase Impedance(volume) above the FRC due to newly recruit volume by HFNC at different flow rate (relative to baseline), which could be reflected by recruited regions(pixels). ΔIrecruit, refer to the product of new pulmonary alveoli recruitment by the increase of airway pressure.

● ΔIaeration, is defined as the increase Impedance(volume) above the FRC due to the increase of airway pressure by HFNC at different flow rate (relative to baseline). ΔIaeration, refer to the product of lung compliance (expansion of the original ventilated alveoli) and the increase of airway pressure.

● ΔEELI, was defined as a total change of end-expiratory lung impedance(volume) than baseline. Hence, ΔIaeration =ΔEELI- ΔIrecruit. Moreover, the ΔIaeration and ΔIrecruit cannot be distinguished directly by EIT.

● IFRC-baseline, was defined impedance(volume) of functional residual capacity(FRC) at baseline.

Hence, the related calculation of lung strain as the following [26]:

● Dynamic lung strain=TV/ (IFRC-baseline+ ΔIrecruit)

● Static lung strain = (ΔEELI− ΔIrecruit)/ (IFRC-baseline+ ΔIrecruit)

● Global lung strain= (TV+ΔEELI− ΔIrecruit,)/ (IFRC-baseline+ ΔIrecruit)

The schematic representation of the effect HFNC on lung strain shown in figure1. Since the absolute value of IFRC-baseline was unknown, the accurate lung strain can-not be calculated. In the present study, ΔIrecruit, ΔIaeration and IFRC-baseline, as the conceptual parameters, were used to indirectly deduct lung strain.


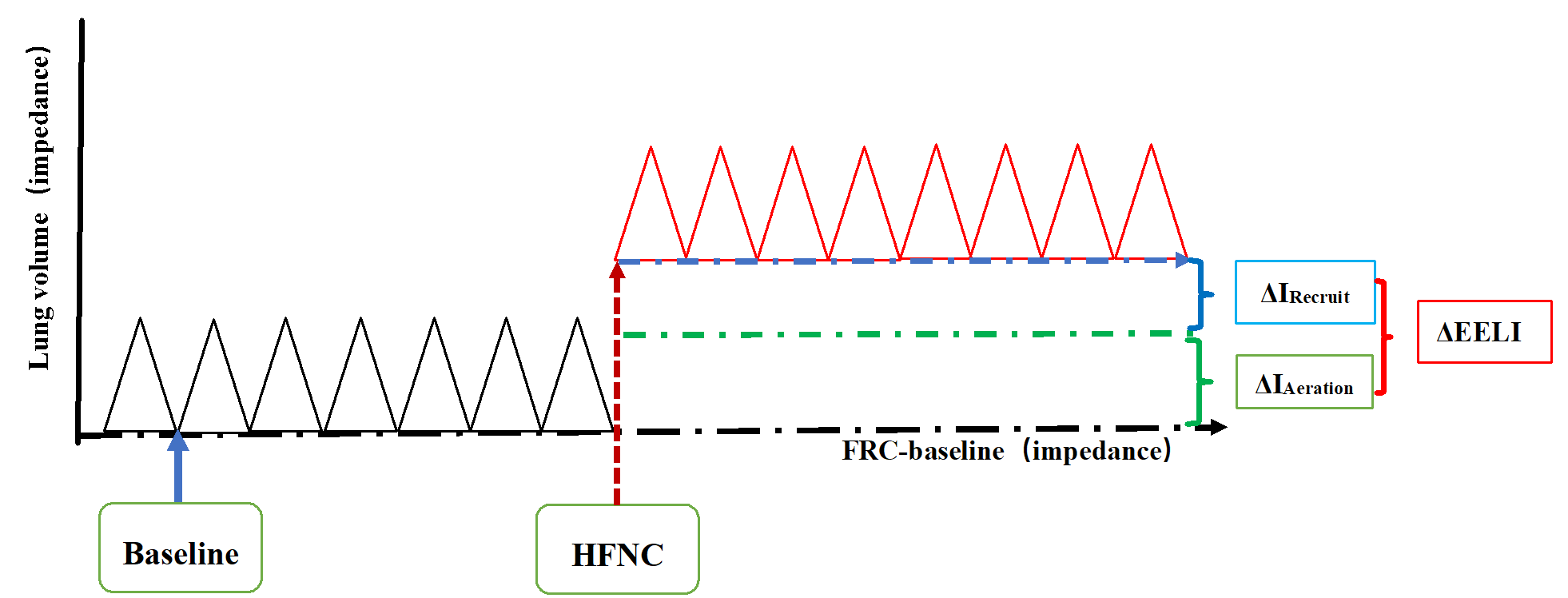


**FigureS1. Schematic representation of the effect HFNC on lung strain.**

1. ΔIrecruit, is defined as the increase volume above the FRC due to newly recruit volume by HFNC at different flow rate (relative to baseline), which could be reflected by recruited regions(pixels). ΔIrecruit, refer to the product of new pulmonary alveoli recruitment by the increase of airway pressure. (2). ΔIaeration, is defined as the increase volume above the FRC due to the increase of airway pressure by HFNC at different flow rate(relative to baseline). ΔIaeration, refer to the product of lung compliance (expansion of the original ventilated alveoli) and the increase of airway pressure. (3). ΔEELI, is defined as a total change of end-expiratory lung impedance than baseline. Hence, ΔIaeration =ΔEELI- ΔIrecruit. Moreover, the ΔIaeration and ΔIrecruit cannot be distinguished directly by EIT. (4) IFRC-baseline, was defined impedance of functional residual capacity(FRC) at baseline.

**The related definition as following:**

 Dynamic lung strain :TV (numerator) unchanged during the study, and we de-fined the IFRC-baseline remained during the study, and the ΔIrecruit (denominator)may be increased(or unchanged)，thus the dynamic lung strain will be de-creased(or unchanged)

 Static lung strain: as the numerator (ΔEELI-ΔIrecruit) may be uncertain or un-changed, and denominator((IFRC-baseline+ΔIrecruit) may be increased or unchanged, thus the result may be diverse (uncertain or unchanged)

 Global lung strain=dynamic lung strain+ static lung strain，thus the result may be diverse (increased, decreased or unchanged)

**Change of lung strain in two groups**

The high potential of recruitment group has a total of 13 recruited-patients without overdistension-_by HFNC_ (equal to a decreased in dynamic lung strain: unchanged TV +increased recruitment region). Moreover, 11 non-recruited patients were in the low potential of recruitment group (in which 4 non-recruited patients with overdistension) (equal to an unchanged in dynamic lung strain: unchanged TV + unchanged recruitment region) (Table S1)

| Variables | High potential of recruitment group  N=13 | | | Low potential of recruitment group  N=11 | | |
| --- | --- | --- | --- | --- | --- | --- |
|  | Baseline | 60L/min | P-value | Baseline | 60L/min | P-value |
| ΔEELI_gl_ (%) | Baseline | 51±56 | 0.042* | Baseline | 51±47 | 0.005* |
| ΔVT_gl_ (%) | Baseline | 7±27 | 1.000 | Baseline | 20±48 | 0.197 |
| Recruitment region  (pixels) | Baseline | 30±22 | 0.002* | Baseline | 3±4 | 0.285 |
| Dynamic lung strain | ↓↓ | | | — | | |
| Static lung strain | ↑/↓↓/— | | | ↑ | | |
| Global lung strain | ↑/↓↓/— | | | ↑/↓/— | | |

**Table S1. Difference of lung strain between high potential of recruitment group and low potential of recruitment group at 60L/min**

ΔEELI=change of end-expiratory lung impedance，ΔVT= change of Tidal Variation，↑=increased，↓=decreased，— = unchanged.

*p<0.05 (Pairwise comparison based on estimated marginal means in the General Linear Model Repeated Measures mode, adjustment for multiple comparison by Bonferroni)

**Reference**

1. Garcia-Prieto E, Lopez-Aguilar J, Parra-Ruiz D, Amado-Rodriguez L, Lopez-Alonso I, Blazquez-Prieto J, Blanch L, Albaiceta GM: Impact of Recruitment on Static and Dynamic Lung Strain in Acute Respiratory Distress Syndrome. Anesthesiology 2016, 124(2):443-452.
2. Aguirre-Bermeo H, Turella M, Bitondo M, Grandjean J, Italiano S, Festa O, Moran I, Mancebo J: Lung volumes and lung volume recruitment in ARDS: a comparison between supine and prone position. Ann Intensive Care 2018, 8(1):25.
3. Chiumello D, Carlesso E, Cadringher P, Caironi P, Valenza F, Polli F, Tallarini F, Cozzi P, Cressoni M, Colombo A et al: Lung stress and strain during mechanical ventilation for acute respiratory distress syndrome. Am J Respir Crit Care Med 2008, 178(4):346-355.
4. Dellamonica J, Lerolle N, Sargentini C, Beduneau G, Di Marco F, Mercat A, Richard JC, Diehl JL, Mancebo J, Rouby JJ et al: PEEP-induced changes in lung volume in acute respira-tory distress syndrome. Two methods to estimate alveolar recruitment. Intensive Care Med 2011, 37(10):1595-1604.
5. Frerichs I, Hinz J, Herrmann P, Weisser G, Hahn G, Dudykevych T, Quintel M, Hellige G: Detection of local lung air content by electrical impedance tomography compared with electron beam CT. J Appl Physiol (1985) 2002, 93(2):660-666.
6. Hinz J, Hahn G, Neumann P, Sydow M, Mohrenweiser P, Hellige G, Burchardi H: End-expiratory lung impedance change enables bedside monitoring of end-expiratory lung vol-ume change. Intensive Care Med 2003, 29(1):37-43.
7. Marquis F, Coulombe N, Costa R, Gagnon H, Guardo R, Skrobik Y: Electrical Imped-ance Tomography's Correlation to Lung Volume is Not Influenced by Anthropometric Pa-rameters. Journal of Clinical Monitoring and Computing 2006, 20(3):201-207.
8. Karsten J, Meier T, Iblher P, Schindler A, Paarmann H, Heinze H: The suitability of EIT to estimate EELV in a clinical trial compared to oxygen wash-in/wash-out technique. Biomed Tech (Berl) 2014, 59(1):59-64.
